# Defining the molecular targets of cerebellar PKG by quantitative (phospho)proteomics in a knock-out mouse model

**DOI:** 10.1186/2050-6511-14-S1-P17

**Published:** 2013-08-29

**Authors:** Eleonora Corradini, Robert Feil, Albert JR Heck, Arjen Scholten

**Affiliations:** 1 Biomolecular Mass Spectrometry and Proteomics, Utrecht University, Utrecht, The Netherlands; 2 Interfakultäres Institut für Biochemie, Universität Tübingen, Tübingen, Germany

## Background

The NO/cGMP/PKG pathway plays a crucial role in the induction of cerebellar long term depression (LTD), synaptic plasticity, and motor learning. Previous studies showed that Purkinje cell specific PKG I knock-out mice exhibit strongly reduced LTD and deficits in adaptation of the vestibular ocular reflex [1].

To better understand the molecular mechanisms of PKG I and its involvement in LTD and motor learning, we combined the use of a mouse model deficient for PKG I expression with an LC-MS/MS based in-depth proteomics and phosphoproteomics analysis directly in cerebellum tissue.

## Results

We compared the protein expression between cerebellum tissues of 3 PKG I deficient and 3 wild-type litter mates. This triplicate analysis yielded a comprehensive cerebellum proteome of 6263 quantified proteins, of which 121 proteins were found to be significantly differentially expressed in the PKG I knock-out mice. Differential expressed proteins included those involved in calcium handling, but also several phosphatases, kinases and phosphodiesterases responded to the absence of PKG.

Next, we also probed changes at phosphorylation level. Therefore, a selective phosphopeptide enrichment strategy was employed. This allowed the identification and quantification of almost 3500 unique phosphopeptides of which a selected set was differentially regulated in the PKGI-deficient mice. These data provides several leads to follow to come to a better understanding of the cerebellum function of of PKG.

## Conclusion

The combination of a transgenic mouse model with an in-depth proteome and phosphoproteome approach provides a powerful platform to understand the molecular biology downstream of PKG I in cerebellum.
